# Autoscopy of the Larynx and the Trachea

**Published:** 1897-02

**Authors:** 


AUTOSCOPY OF THE LARYNX AND THE TRACHEA. (Direct examination without mirror.) By Alfred Kirstein, M. D., Berlin. Authorized translation (altered, enlarged, and revised by the author) by Max Thorner, A. M., M. D., Cincinnati, Ohio, Professor of Clinical Laryngology and Otology, Cincinnati College of Medicine and Surgery; Laryngologist and Aurist, Cincinnati Hospital, etc. With twelve illustrations. One volume, crown octavo, pages xi–68. Extra cloth, 75 cents, net. The F. A. Davis Co., Publishers, 1914 and 1916 Cherry Street, Philadelphia; 117 West Forty-second Street, New York; 9 Lakeside Building, Chicago.

This interesting little monograph describes an improved method of examining the larynx without the use of a mirror. All physicians naturally take for granted that it is absolutely necessary to use a mirror in examinations of the throat because of the evident fact that the light must turn an angle in coming to the eye.

The author of this book shows that this general opinion is not strictly true. He describes the method of placing the body and depressing the tongue so that a straight line is formed from the vocal cords to the observer's eye, and then with a small incandescent electric lamp of about the size of a pea and having a half-candle power, a sufficient illumination is produced to enable the operator to look directly at the vocal cords and into the larynx even as far down as the sixth ring, and that too without the inversion and consequent confusion of viewing the image in a mirror.

The method of accomplishing this admirable result is told with great clearness and detail in this little book, and thus it is an indispensable adjunct to the library of every physician who contemplates accurate examination of the larynx. It is even possible, with this apparatus, to photograph the larynx; and this would be valuable as a record for the practical laryngologist.

